# Variations in COVID-19 Vaccine Attitudes and Acceptance among Refugees and Lebanese Nationals Pre- and Post-Vaccine Rollout in Lebanon

**DOI:** 10.3390/vaccines10091533

**Published:** 2022-09-15

**Authors:** Zawar Ali, Shiromi M. Perera, Stephanie C. Garbern, Elsie Abou Diwan, Alaa Othman, Javed Ali, Nada Awada

**Affiliations:** 1 International Medical Corps, Washington, DC 20036, USA; 2 Department of Emergency Medicine, Alpert Medical School of Brown University, Providence, RI 02903, USA; 3 International Medical Corps, Beirut 362060, Lebanon

**Keywords:** COVID-19, SARS-CoV2, coronavirus, pandemic, Lebanon, vaccines, vaccine hesitancy, epidemic, outbreak

## Abstract

Vaccine hesitancy among displaced populations is associated with inequitable access to services and mistrust of authorities, among other factors. This study evaluated variations in attitudes toward COVID-19 vaccines and factors associated with vaccine acceptance among refugees and Lebanese nationals accessing 60 International Medical Corps-supported health facilities through two cross-sectional surveys pre- (*n* = 3927; Survey 1) and post- (*n* = 4174; Survey 2) vaccine rollout. Logistic regression was used to assess predictors of vaccine acceptance using the health beliefs model. Refugees comprised 52.9% (Survey 1) and 54.2% (Survey 2) of respondents. Vaccine acceptance was low among both groups in Survey 1 (25.9% refugees vs. 23.1% Lebanese nationals), but higher in Survey 2 in Lebanese (57.6%) versus refugees (32.9%). Participants reported greater perceived benefits of vaccination, higher perceived COVID-19 susceptibility, and lower perceived vaccination barriers in Survey 2 versus Survey 1. Post-vaccine rollout, refugees had lower odds of vaccine acceptance compared to Lebanese (OR 0.50, 95%CI 0.41–0.60), while older age (OR 1.37, 95%CI 1.06–1.78, ≥51 years vs. 18–30 years) was associated with greater vaccine acceptance. Health beliefs model variables were associated with vaccine acceptance in both surveys. Tailored strategies to respond dynamically to changes in vaccine attitudes among vulnerable groups in Lebanon are essential for equitable vaccine uptake.

## 1. Introduction

The COVID-19 pandemic has led to an unprecedented global health crisis, with over 587 million cases of COVID-19 and 6.4 million deaths reported as of August 2022 [1]. While more than 12.4 billion vaccine doses have been administered worldwide, large disparities in vaccine access and uptake exist [2]. Notably, only 20.7% of people in low-income countries have received at least one dose of vaccine compared to 67.4% globally, and 78.5% in high-income countries [2]. This disparity is further exacerbated in humanitarian settings among disaster and conflict-affected populations, where additional unique challenges exist related to vaccine access and logistics, mistrust in authorities, insecurity, weak health infrastructure, as well as vaccine hesitancy concerns specific to refugees and marginalized populations [3].

Vaccine hesitancy, as defined by the World Health Organization (WHO) Strategic Advisory Group of Experts on Immunization (SAGE) Working Group, is the “delay in acceptance or refusal of vaccines despite availability of vaccine services” [4]. Vaccine hesitancy has been shown to be rising globally among diverse populations and is both complex and context specific, varying across time, place, and vaccines, as well as being influenced by factors such as complacency, convenience, and confidence [4].

Lebanon has 1.2 million confirmed cases of COVID-19 and 10,576 reported deaths as of August 2022 [5]. The outbreak occurred at a time when Lebanon was already facing multiple crises and challenges: an unprecedented financial and sociopolitical crisis; the August 2020 explosion at the Port of Beirut, which destroyed three hospitals and damaged another three; and an already fragile healthcare system, due to the protracted Syrian refugee crisis [6,7]. Lebanon has had several large influxes of refugees over the past 74 years, starting with Palestinians who migrated during the 1948 and 1967 Palestine wars with Israel, and more recently, Syrians who have migrated since the Syrian civil war began in 2011 [8,9]. As a result, Lebanon hosts the highest refugee population per capita in the world, with one-third of its 6.8 million population comprised of 1.5 million Syrians, 400,000 Palestinians, and smaller numbers from Iraq, Sudan, and Ethiopia [6].

Even before the pandemic, refugees in Lebanon faced poor health and socioeconomic status as a consequence of tenuous legal status, poverty, reduced access to healthcare, and poor-quality accommodations [10,11]. Restrictive residency regulations have left many refugees without lawful status, making it difficult to acquire work permits, leading to informal employment that has resulted in many living below poverty level [11]. Only 20% of Syrians have legal residency and 90% of Syrian families live in extreme poverty [9,12]. Refugees, especially Syrians, have difficulty in accessing healthcare services, resulting in the reliance on informal unlicensed healthcare workers [13]. Refugees and vulnerable Lebanese nationals also have access to a network of national primary healthcare centers (PHCCs) around the country that are run by the Ministry of Public Health (MoPH), municipalities, and non-governmental organizations (NGOs) at a nominal fee, which is further subsidized for refugees [14]. While these centers provide a comprehensive package of primary care services, the system has experienced issues related to the impact of the refugee crisis, funding, urban–rural disparities in staffing, and infrastructure [14,15]. Syrian refugees, in particular, constitute 47% of all those who access care through the PHCCs; however, uptake of certain health services pre-pandemic, including routine immunization, was found to be lower among refugees compared to Lebanese nationals [15,16]. By one estimate from a 2015 survey of 1400 Syrian refugee households, only 12.5% of children aged 12–23 months were fully immunized [17].

There is evidence that the pandemic has further worsened these conditions by disproportionately affecting the refugee population in Lebanon. Loss of jobs during the pandemic and the inability to pay for medicine and health care are a reality for most refugee families [18,19,20]. In early 2021, refugees in Lebanon had a COVID-19 fatality rate that was 3–4 times the national average [19,20]. Considerable inequities in access to vaccines among refugees in Lebanon have been reported, even though free access was guaranteed to all nationalities and vaccination plans currently target all people above age 11 [6,19,21,22]. Since COVID-19 vaccines were first rolled out in Lebanon in February 2021, a total of 5.7 million COVID-19 vaccine doses have been administered as of August 2022. Among Syrian and Palestinian refugees, only 13% are vaccinated and 18% have registered on the national platform to receive a vaccine [5,23]. However, this is not unique to Lebanon. During pandemics, displaced populations tend to shoulder a disproportionate burden of disease due to difficulties with obtaining access to high-quality healthcare, economic hardships, mental illness and de-prioritization during times of severe resource constraints [3,24].

One study of Lebanese adults conducted prior to vaccine rollout found 21.4% would accept a COVID-19 vaccine, with higher vaccine hesitancy found among women, married participants, and those who had greater hesitancy towards vaccines in general [25]. An online survey of Lebanese adults in February 2021 reported a COVID-19 vaccine acceptance rate of 63.4%, and multivariable analysis showed that higher knowledge of COVID-19 vaccines, living in an urban area, and greater fear of COVID-19 infection were positive predictors of acceptance. However, this higher vaccine acceptance rate may have been overestimated due to respondents being predominantly highly educated [26]. Another study conducted between January and February 2021, of older Syrian refugees, found that 28.8% reported no intention to vaccinate. Vaccine refusal was significantly associated with perceptions of vaccine safety and effectiveness, especially related to the newness of the vaccine [27]. However, there remains a paucity of research on vaccine hesitancy among refugee and host community populations in Lebanon, and on how vaccine attitudes have changed in relation to vaccine rollout in this setting. The aim of this study was to evaluate the knowledge, attitudes, and perceptions of adults who accessed IMC services, specifically refugees and Lebanese nationals of low socioeconomic status accessing IMC services regarding COVID-19 vaccines, and assess factors associated with vaccine acceptance pre- and post-vaccine rollout.

## 2. Materials and Methods

### 2.1. Study Design and Setting

Two cross-sectional surveys were conducted by International Medical Corps (IMC), in collaboration with Lebanon’s Ministry of Public Health (MOPH), among adults seeking services at one of 60 IMC-supported national primary health care centers (PHCC) as part of IMC’s routine operations. The PHCCs support refugees and Lebanese nationals with low socioeconomic status. The first survey was conducted in February 2021, just prior to COVID-19 vaccine rollout, and a second survey was conducted in June 2021, 4 months after vaccine rollout.

IMC has been working in Lebanon since 2006, expanding its role following the onset of the 2011 Syrian crisis with programming to meet the basic health needs of refugees and reducing the gap in vaccine coverage by supporting a network of 60 PHCCs and dispensaries across the country. As one of the key partners in the national COVID-19 Risk Communication and Community Engagement (RCCE) and accountability taskforce, IMC, together with over 40 other government and non-government organizations, has been working to increase COVID-19 vaccine acceptance and uptake through various initiatives, including communication and programming interventions.

As this study only used anonymously collected survey data, formal IRB approval was waived by the MOPH of Lebanon (Reference 593 P.H.C.). Vaccines available in Lebanon at the time of this study included: Pfizer-BioNTech, AstraZeneca, Gam-COVID-Vac (Sputnik V), Sinopharm, Moderna, and Johnson & Johnson [23]. The PHCCs surveyed in this study are located in the following 6 governorates: Beirut (5 PHCCs), Akkar (10 PHCCs), Bekaa (13 PHCCs), Mount Lebanon (10 PHCCs), North (9 PHCCs), and South (10 PHCCs). Beirut and Tripoli are urban areas, while the Mount Lebanon and South governorates are a mix of urban and rural. Bekaa, Koura District of the North governorate, and Akkar are rural and underserved areas.

### 2.2. Study Population

Any adult (18 years or older) receiving services at an IMC-supported PHCC was eligible for participation. Both surveys were conducted in the same catchment area. A convenience sample was employed using IMC’s database of adult participants in the PHC program for each area. Also, adults seeking services at the PHCC were offered participation in the survey. Participants are registered in the PHCC database as either refugees (non- Lebanese) or Lebanese nationals. An equal number of male and female respondents, and equal numbers of refugees and Lebanese nationals were selected.

### 2.3. Survey Instrument

Demographic data on age (categorized as 18–30 years, 31–50 years, and 51 years or older), gender, and nationality were collected. In Survey 1, nationality data was reported as Lebanese national vs. refugees, while in Survey 2, nationality data was reported as Lebanese national, Syrian, Palestinian, or Other. The survey was adapted from the list of questions of the Strategic Advisory Group of Experts on Immunization (SAGE) vaccine hesitancy matrix by WHO [28]. Apart from directly asking about the vaccine acceptance and whether the respondents had registered with the government for vaccination, the questions were based on the following health beliefs model (HBM) domains: perceived susceptibility, perceived severity, perceived benefit, perceived barriers, cues to action, and social norms. The survey was created in Google Forms and conducted by interviewers with answers recorded on tablets. Interviews were conducted in Arabic since both the Lebanese nationals and refugee respondents speak a similar Levantine Arabic language dialect. Interviewers were trained to use the survey instrument, how to interview participants, and the informed consent process. Interviews were conducted face to face or over the phone, depending on their level of accessibility due to COVID-19 measures.

### 2.4. Survey Items and Health Beliefs Model Components

The independent variables included sociodemographic variables (age category, gender, nationality), knowledge about the COVID-19 vaccine (6 items, Cronbach’s α = 0.67), social norms (1 item), and the 5 HBM domains. Principal components exploratory factor analysis using varimax rotation was conducted on the survey items to create scales according to the health belief model items. Rotated factor loadings of ≥ |0.4| were accepted. Cronbach’s alpha was used to evaluate the internal validity (reliability) of the survey and item scales. Negative items were reverse scored so that higher scores indicated higher levels of the item. The 5 HBM domains included perceived susceptibility (2 items, Cronbach’s α = 0.67), perceived severity (1 item), perceived barriers (5 items, Cronbach’s α = 0.79), perceived benefits (2 items, Cronbach’s α = 0.85), and cues to action (1 item). Cronbach α for survey was 0.82, indicating excellent internal consistency. Multicollinearity among the predictors was assessed using variance inflation factor (VIF) with VIF ≤ 5 indicating a lack of multicollinearity.

### 2.5. Primary Outcome

The primary outcome was defined as intent to receive a COVID-19 vaccine. This survey item had three possible responses (“yes”, “no”, “unsure”). For this analysis, COVID-19 “vaccine acceptance” was defined as having a “yes” response to the question “Will you receive the COVID-19 vaccine when it is available in Lebanon?”; “vaccine hesitancy” was categorized having a response of either “no” or “unsure”, according to the SAGE Working Group definition of vaccine hesitancy as “delay in acceptance or refusal of vaccination despite the availability of vaccination services.”

### 2.6. Data Analysis

Descriptive analysis was conducted using frequencies with percentages or medians with interquartile ranges (IQR) as appropriate. Data were stratified and comparisons conducted with Mann–Whitney U test or Pearson’s chi-squared test as appropriate to evaluate for associations between intent to receive a COVID-19 vaccine with respect to age, gender, nationality, and survey timepoint (i.e., Survey 1 in February 2021 vs. Survey 2 in June 2021).

Multivariable logistic regression was performed to assess for associations between the independent variables and the primary outcome (intent to receive a COVID-19 vaccine) with magnitudes of effect given as adjusted odds ratios (OR) and their respective 95% confidence intervals (CI). Model discrimination was calculated using area under the receiving operator characteristic (ROC) curve (AUC). Nagelkerke’s pseudo R^2^ was calculated to provide a global measure of the estimated explained variance of the model. For all analyses, a two-tailed *p*-value of 0.05 was considered statistically significant. STATA Version 16 (Stata Corp; College Station, TX, USA) was used for all analyses.

## 3. Results

### 3.1. Participant Characteristics

A total of 3927 participants completed Survey 1 (February 2021) and 4174 participants completed Survey 2 (June 2021); descriptive characteristics of the study populations are shown in Table 1. There were slightly more female participants in both surveys (54.5% in Survey 1 and 54.2% in Survey 2). Participants in Survey 2 were slightly older (30.1% in Survey 2 vs. 23.5% in Survey 1 were ≥51 years) as shown in Table 1. Slightly over half of the participants in both surveys were refugees (52.9% in Survey 1 and 53.3% in Survey 2). Of those that reported they were refugees, 95.7% were Syrian.

### 3.2. Vaccine Acceptance

Vaccine acceptance (i.e., intention to receive a COVID-19 vaccine or having received a vaccine) was higher in Survey 2 than Survey 1. In Survey 1, 959 (24.4%) of participants intended to receive a vaccine, while 985 (25.1%) were unsure, and 1983 (50.5%) would refuse. In Survey 2, 1854 (44.4%) intended to or had received a vaccine (16.9% had received a vaccine), while 986 (23.6%) were unsure, and 1334 (32.0%) would refuse (Survey 1 vs. Survey 2, *p* < 0.001).

**Figure 1:**
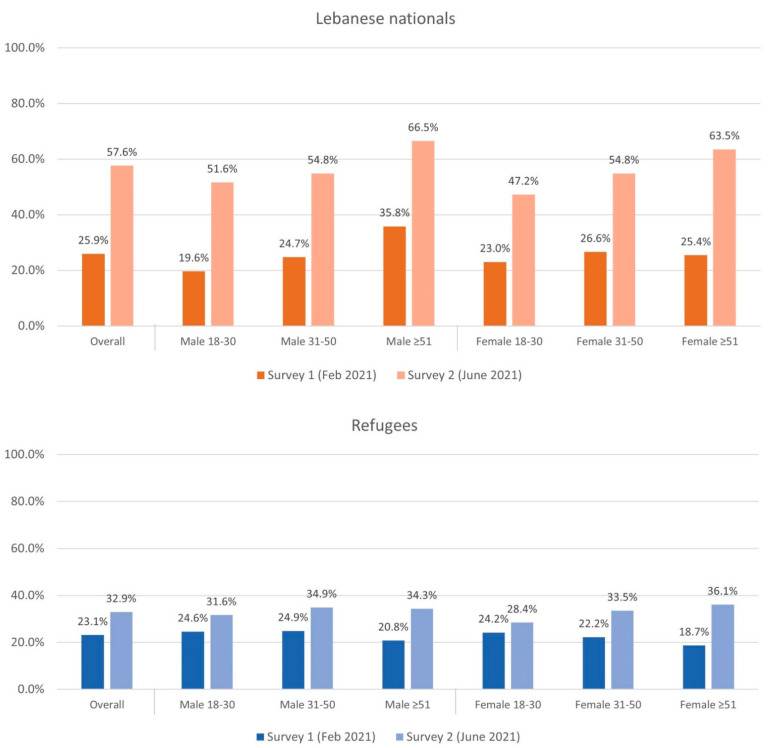
Vaccine intention among Lebanese nationals and refugee participants grouped by age category and gender in Survey 1 and Survey 2.

Vaccine acceptance was also stratified by age category, gender, and nationality. In Survey 1, there was no association between age and vaccine acceptance; however, in Survey 2, vaccine acceptance was higher among older age categories (53.9% in 51 or older, 42.5% in 31–50 years, and 37.2% in 18–30 years). Regarding gender, in Survey 1, there was no association between gender and vaccine acceptance (25.4% in male vs. 23.6% in female, *p* = 0.08); however, in Survey 2, males had higher vaccine acceptance (46.2% in male vs. 42.9% in female, *p* = 0.022). Regarding nationality, in Survey 1, Lebanese were slightly more likely to intend to receive the vaccine compared to refugees, although this did not reach statistical significance (25.9% Lebanese vs. 23.1% refugees, *p* = 0.053); however, in Survey 2, Lebanese were significantly more likely to have vaccine acceptance (57.6% of Lebanese vs. 32.9% of refugees, *p* < 0.001). Vaccine intention grouped by age, gender, and nationality for both surveys are shown in Figure 1.

### 3.3. Health Beliefs Model (HBM) Domains and Social Norms

Participants showed greater perceived benefit, perceived susceptibility and severity, and lower perceived barriers in Survey 1 compared to Survey 2 (Table 2). Mean scores for the HBM domains by intention to receive a vaccine in Survey 1 and Survey 2 are shown in Table 2. When comparing participants by nationality, refugees had lower perceived susceptibility and perceived severity of COVID-19 compared to Lebanese participants in both surveys. Refugees more often reported that they would not take the vaccine because they were not at risk of severe complications (53.6% of refugees vs. 42.3% of Lebanese in Survey 1 and 41.4% of refugees vs. 25.3% of Lebanese in Survey 2) and because they were in good health (51.4% of refugees vs. 38.5% of Lebanese in Survey 1 and 42.2% of refugees vs. 23.9% of Lebanese in Survey 2).

By Survey 2, confidence increased in vaccine safety (15.3% of refugees vs. 15.7% of Lebanese in Survey 1 and 31.1% of refugees vs. 46.5% of Lebanese in Survey 2) and efficacy (14.8% of refugees vs. 16.8% of Lebanese in Survey 1 and 29.3% of refugees vs. 42.9% of Lebanese in Survey 2), especially among Lebanese participants. The percentage of participants that were concerned with the quick development of the vaccine and its side effects had also reduced by Survey 2. Comparisons between refugees and Lebanese participants are shown in Supplemental Table S2 (Survey 1) and Table S3 (Survey 2).

Regarding social norms, the gap between Lebanese and refugees widened between Survey 1 and Survey 2. In Survey 1, 12.4% of Lebanese and 12.7% of refugees reported most people they knew were going to receive the vaccine, compared to 30.5% of Lebanese vs. 14.6% of refugees in Survey 2.

### 3.4. Knowledge, Attitudes, and Beliefs

Knowledge regarding the COVID-19 vaccines was higher in Survey 2 than in Survey 1 (Table 3). Most participants reported knowing there were different COVID-19 vaccines (55.3% in Survey 1 and 85.1% in Survey 2, *p* < 0.001), having enough information about the vaccines/how they work (23.3% in Survey 1 and 65% in Survey 2, *p* < 0.001) knowing the vaccine is available for all nationalities and free of charge (51.8% in Survey 1 and 89.4% in Survey 2, *p* < 0.001), and being aware of the eligibility categories and prioritization (64.5% in Survey 1 and 46.5% in Survey 2, *p* < 0.001). While only asked in Survey 2, only 27.9% believed the vaccine could be given to pregnant women, while 29.0% were unsure, and 43.2% believed it could not be given to pregnant women.

When asked “Do you think there is a better way than the vaccine to fight COVID-19?”, 23.4% of participants said “yes” in Survey 1. These participants were asked what other ways they could fight COVID-19. They stated several prevention and protection measures, such as: social distancing (*n* = 97, 17.5%); boosting their immunity with the help of vitamins, herbal drinks, and healthy foods (ex: ginger, lemon, garlic, or onion) (*n* = 93, 16.8%); hygiene practices (*n* = 73, 13.2%); wearing masks (*n* = 65, 11.7%); quarantine (*n* = 71, 12.8%); avoiding crowds by staying at home (*n* = 37, 6.7%); or entrusting their soul to Allah (*n* = 19, 3.4%). By Survey 2, fewer participants (15.8%) thought there was a better method than the vaccine, with most of them stating preventive and protective measures, rather than common myths.

Attitudes and beliefs toward the vaccines are shown in Table 3. In Survey 1, 21.4% reported it was possible they would accept one vaccine and refuse another, compared to 47.6% in Survey 2. Fewer participants reported they would wait until vaccines are proven safe in Survey 2 compared to Survey 1 (68.8% in Survey 1 vs. 53.9% in Survey 2). Half (50.2%; 47.0% of refugees vs. 53.8% of Lebanese) of the participants in Survey 2 reported confidence that the Lebanese health system could ensure safe administration of the COVID-19 vaccine, compared to 36.5% (39.2% of refugees vs. 33.4% of Lebanese) in Survey 1.

### 3.5. Communication

Regarding misinformation and disinformation, one-third (33.3%) of the overall population in Survey 1 and 21.7% in Survey 2 responded “yes” to “Have you heard a lot of false or negative information about the COVID-19 vaccines?” Participants that answered “yes” reported hearing about how the vaccine leads to death or disability (*n* = 75, 43.6%), has side effects (*n* = 35, 20.3%), causes infertility or affects hormones (*n* = 10, 5.8%), and changes DNA (*n* = 6, 3.5%).

**Figure 2:**
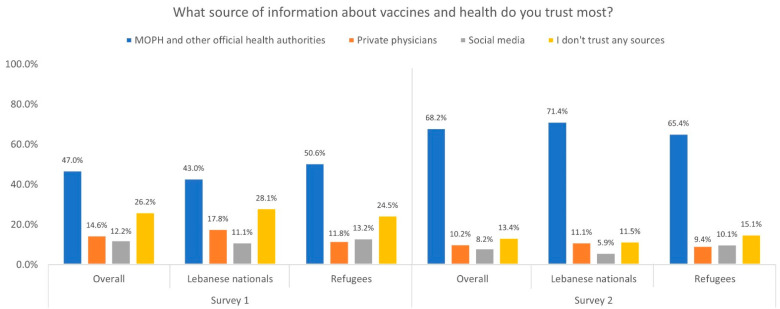
Most trusted sources of information regarding vaccines and health among overall study population and by nationality in Survey 1 and Survey 2.

The most trusted sources of information about vaccines and health reported by participants are shown in Figure 2. The MOPH and official health authorities were the most trusted source of information in both surveys, in the overall study population, as well as among both Lebanese and refugee participants (47% in Survey 1 and 68.2% in Survey 2). Participants also relied on private physicians (14.6% in Survey 1 and 10.2% in Survey 2) or social media sources (12.2% in Survey 1 and 8.2% in Survey 2).

### 3.6. Barriers to Vaccination and Preferences for Vaccination Sites

In Survey 1, the most common barriers to vaccination reported by respondents include: a difficult registration process (19.7%), security issues (17.8%), and issues related to transportation to the vaccine site/site being too far (17.5%). Only 15.1% reported “no barriers.” Barriers were much less commonly reported in Survey 2: only 8.7% reported a difficult registration process, 7.8% reported transportation issues, and 1.8% reported security issues, with 74.2% reporting “no barriers.” Registration on the MOPH platform increased between Survey 1 and Survey 2, although the gap between Lebanese and refugees widened, with 10.3% of overall participants (12.2% of Lebanese, 8.7% of refugees) having registered in Survey 1, compared to 40.0% (54.4% of Lebanese vs. 27.3% of refugees) of participants in Survey 2 (*p* < 0.001).

For preferences for vaccination sites, in Survey 1, 37.3% of the overall study population preferred to receive the vaccine in MOPH vaccination centers, followed by in primary health centers or dispensaries (32.7%), and then private clinics, even if the vaccine was not free (19.0%). In Survey 2, the same order of preferences was noted, with higher prevalence of plans to receive the vaccine in MOPH centers (58.7%), primary health centers (40.3%), at their residence/home (29.3%, only asked in Survey 2), and similar numbers to Survey 1 for private clinics (19.2%). Results stratified by nationality (Lebanese vs. refugees) for both surveys are shown in Table 4.

### 3.7. Regression

In multivariable analysis, in Survey 1, gender, nationality, and age were not found to be associated with intention to receive a COVID-19 vaccine. However, in Survey 2, older age and Lebanese nationality were associated with greater odds of intending to receive a vaccine, while younger individuals and refugees were associated with lower odds. Univariate analyses for both surveys are shown in Supplemental Table S1. All HBM domains except for perceived severity were associated with the primary outcome in both surveys (Table 5). Having higher perceived benefit, perceived susceptibility and severity, cues to action, and social norms were associated with vaccine acceptance, while those with higher perceived barriers were associated with lower odds of vaccine acceptance. However, in Survey 2, having greater perceived severity was associated with having greater odds of vaccine acceptance, although it was marginally insignificant (OR 1.27 95%CI 1.00–1.61). While having greater knowledge of COVID-19 vaccines and eligibility was associated with greater intention to receive a vaccine in Survey 1, this was not associated in Survey 2. The AUC of Survey 1 was 0.929, and that of Survey 2 was 0.933, indicating excellent ability to discriminate between intention to receive a vaccine or not using these models. The pseudo R^2^ was 0.642 in Survey 1 and 0.684 in Survey 2, indicating that the models explained 64.2% and 68.4% of the variability.

## 4. Discussion

Understanding and addressing factors that influence vaccine acceptance and uptake are critical to improving equity in vaccine coverage and controlling COVID-19 in Lebanon. This study evaluated knowledge, attitudes, and perceptions towards the COVID-19 vaccines and factors associated with COVID-19 vaccine acceptance among adults seeking services from a non-profit humanitarian organization operating in Lebanon. A strength of this study is the recruitment of a large group of highly vulnerable individuals including both Lebanese nationals and refugee PHCC participants, and the collection of data at two key timepoints in the pandemic, just before and several months after the rollout of vaccines widely in Lebanon.

In our study, we utilized the health beliefs model (HBM) to identify sociodemographic characteristics and demand side factors associated with vaccine acceptance. The HBM model has previously been shown to be a highly useful model for childhood and adult vaccine acceptance in diverse groups, even before COVID-19 [29,30,31,32,33,34]. Since the COVID-19 pandemic, the model has been used extensively to study and predict factors associated with COVID-19 vaccine acceptance [35,36,37,38,39,40,41,42,43]. The robust discrimination and pseudo R^2^ showed that the model explained a majority of the variability in individuals’ willingness to take a COVID-19 vaccine, needed to understand the main drivers of vaccine acceptance and hesitancy in these populations.

Prior to vaccine rollout, our study found that only one-quarter of participants were accepting of COVID-19 vaccines. Other studies from the Middle East have assessed vaccine acceptance in different contexts. A cross-sectional online study from Arab countries, including Jordan, Kuwait, and Saudi Arabia, showed around 29% acceptance rate in their sample, with slight difference across countries [44]. In addition, vaccine acceptance was higher in males, people who had a history of chronic diseases, and people with post-graduate education [44]. However, there are other studies from Arab countries in which the acceptance rates have been higher; an internet-based survey in the United Arab Emirates showed 60% acceptance in September 2020, while another online survey, conducted in December 2020 and January 2021 in 22 Arab League countries, showed a 62% acceptance rate [42,43]. Our study found that in Survey 2, the acceptance rates after vaccine rollout were notably higher; this increase in vaccine acceptance was anticipated due to public awareness campaigns and communication activities being conducted according to the Risk Communication and Community Engagement (RCCE) plan by the Ministry of Public Health (MOPH) shortly after the first survey was conducted [6].

Importantly, an increase in COVID-19 vaccine acceptance was seen primarily among the Lebanese national respondents; this was less pronounced among refugee respondents. Regarding the influence of nationality on vaccine acceptance, a study from UAE showed non-Emirati nationality to be associated positively with COVID-19 vaccine acceptance [45]. Another online survey from Arabic-speaking countries showed higher association between vaccine acceptance and nationality from a high-income country [46]. A strength of our study is the recruitment of both Lebanese and refugees of similar socioeconomic status, living in the same area, and utilizing the same health system. Despite COVID-19 vaccine communication campaigns being in Arabic, the language common to both groups, and targeting the same population, our findings show hesitancy persisted after vaccine rollout to a much greater degree among the refugee respondents. Greater COVID-19 vaccine confidence was also seen in older age categories and in males post-vaccine rollout (although this association was not seen pre-vaccine rollout), indicating that vaccine attitudes shifted among certain groups more than others. Male gender has similarly been shown to be significantly associated with COVID-19 vaccine acceptance in other studies from the region [44,45,46]. Older age was associated with vaccine acceptance in our study sample; this may be related to greater perceived susceptibility in older respondents and lower complacency. Association of vaccine acceptance with age is not clearly established in the literature from the region, and other studies have not found a similar association between age and COVID-19 vaccine acceptance [46,47]. Conversely, a study conducted in the UAE found that vaccine acceptance corelated positively with younger age groups [45].

Amongst the HBM domains, perceived benefits had the strongest positive association with vaccine acceptance, followed by cues to action. Other factors, such as perceived susceptibility and social norms, corelated positively with intention to vaccinate. These findings are in line with other studies using the HBM for understanding vaccine hesitancy, showing that positive motivations (such as desire to protect oneself and one’s contacts) and social influences are strong drivers of vaccine acceptance and uptake [40,45,48,49]. However, perceived barriers corelated negatively with intention to vaccinate. The barriers included in this survey largely focused on perceived dangers of the vaccines, including myths and disinformation regarding the vaccines, as well as logistical barriers, such as difficult registration processes and security issues. These findings are also in line with recent research, mostly from China and US, on vaccine hesitancy/acceptance for COVID-19 vaccines [36,43,50,51].

Vaccine acceptance has been shown to vary across different demographic and socioeconomic groups [45,46,47]. Public health organizations should identify groups with high levels of vaccine hesitancy in order to create targeted interventions when designing plans to increase vaccine confidence. Belief in having susceptibility to COVID-19, high perception of benefits of COVID-19 vaccines, and cues to action consistently show a high association with vaccine acceptance. Vaccine communication should incorporate messaging that focuses on these domains in an effective way. The ability to reach and motivate high hesitancy groups with focused messaging will likely increase vaccine confidence in the target audience.

Interestingly, higher levels of vaccine knowledge did not corelate with vaccine acceptance, despite overall COVID-19 vaccine-related knowledge being higher in Survey 2 compared to Survey 1. An online experiment conducted in UK in April 2021 also suggests that simply aiming to increase vaccine knowledge may not be sufficient to increase vaccine uptake, without addressing other factors that influence decision making [52]

The Lebanese government started a phased COVID-19 vaccine rollout in February 2021, with vaccine hesitancy identified as a major obstacle from the start [53]. The MOPH included all groups, regardless of nationality, in an inclusive vaccination plan to ensure that refugees residing in Lebanon (including those without any formal registration) could access COVID-19 vaccines. However, by January 2022, only 15% of administered doses were given to refugees, falling short of an equitable target of 20% [23]. To ensure vaccine equity, it is critical to create demand and address hesitancy in vulnerable groups. Our study provides evidence that while the RCCE framework has worked well in addressing hesitancy in Lebanese nationals, it has been less effective in refugee communities. Tailored strategies for refugees specifically focusing on reducing concerns about vaccine side effects and combatting disinformation are needed, as is more research into drivers for the lower perceived susceptibility to COVID-19 among the refugee communities in this study. After this survey was conducted, IMC launched a “vaccine hero campaign” in the target population, in which vaccinated members of the refugee community who had a positive perception of vaccines shared their experiences and motivation for vaccination with the community. An impact evaluation of these interventions is currently being conducted. Monitoring vaccine hesitancy, including refugee representatives during the design phase of vaccine rollout, and identifying the most effective strategies to foster demand, will ensure equitable uptake of COVID-19 vaccination in these populations.

## 5. Limitations

Since this study used convenience sampling, these findings cannot be generalized to the general population of Lebanon. However, these findings are still informative and should be used to appropriately tailor COVID-19-related messaging and programming among these communities. The survey tool that was developed was based on the SAGE questionnaire on general vaccine hesitancy, with some additional elements added, which has not been specifically validated for COVID-19 vaccination. Other studies have found that education level, socioeconomic status, and attitudes towards other vaccines and routine immunizations may be associated with COVID-19 vaccine attitudes. However, given the retrospective nature of this study, these data for these additional variables were not available, which makes this a major limitation.

## 6. Conclusions

Ensuring vulnerable groups receive equitable access to COVID-19 vaccines is critical to controlling COVID-19 in Lebanon and globally. Our study highlighted differences in vaccine acceptance among refugees and Lebanese nationals, during two key timepoints in vaccine rollout. These findings suggest the need for more focused, dynamic, and tailored strategies to promote vaccine acceptance, reduce vaccine hesitancy, and ensure vaccine equity for refugees. Strategies should not only focus on knowledge, but also target the individual perceptions towards vaccines, such as their perceived susceptibility and social norms. Additionally, interventions to address perceived barriers to vaccination, such as reducing fears of side effects and combatting disinformation, will also improve confidence in vaccines and the health system that administers it.

## Figures and Tables

**Table 1:** Participant characteristics.

	Survey 1 (*n* = 3927) *n* (%)	Survey 2 (*n* = 4174) *n* (%)	*p* *
**Age Category**			<0.001
18–30 years	1436 (36.6)	1222 (29.3)	
31–50 years	1567 (39.9)	1695 (40.6)	
≥51 years	924 (23.5)	1257 (30.1)	
**Gender**			0.769
Female	2140 (54.5)	2261 (54.2)	
Male	1787 (45.5)	1913 (45.8)	
**Nationality**			0.724
Lebanese	1850 (47.1)	1950 (46.7)	
Refugees	2077 (52.9)	2224 (53.3)	
Palestinian	-	86 (2.1)	
Syrian	-	2129 (51.0)	
Other	-	9 (0.2)	
**Location**			
Akkar	545 (13.9)	884 (21.2)	
Beirut	713 (18.2)	395 (9.5)	
Bekaa	878 (22.4)	743 (17.8)	
Deddeh lkoura	1 (0.03)	-	
Mount Lebanon	580 (14.8)	439 (10.5)	
North Tripoli	453 (11.5)	624 (14.9)	
South	750 (19.1)	1089 (26.1)	
*Missing*	7 (0.2)	-	

* Chi-square.

**Table 2:** Items, response scales, and internal consistency for HBM domains and knowledge scale.

	Intention to Receive Vaccine				
	Survey 1		Survey 2		
Domain	Yes Mean (SD)	Unsure/No Mean (SD)	Yes Mean (SD)	Unsure/No Mean (SD)	*p* *
**Perceived Susceptibility (α = 0.67)**					
I do not need to receive the vaccine because I have good health	0.64 (0.83)	1.31 (0.81)	0.31 (0.66)	1.24 (0.86)	<0.001
I do not need to receive the vaccine if had been infected with COVID-19 and recovered	0.62 (0.79)	1.07 (0.80)	0.38 (0.68)	0.94 (0.84)	<0.001
**Perceived Severity**					
I am not at risk of severe complications of COVID-19 so I will not take the vaccine (Yes/No)					<0.001
Yes	1603 (54.0)	294 (30.7)	1133 (48.8)	281 (15.2)	
No	1365 (46.0)	665 (69.3)	1187 (51.2)	1573 (84.8)	
**Perceived Benefit (α = 0.85)**					
I think the COVID-19 vaccine is safe	1.48 (0.60)	0.58 (0.56)	1.70 (0.49)	0.85 (0.61)	<0.001
I think the COVID-19 vaccine is effective	1.48 (0.57)	0.66 (0.55)	1.64 (0.51)	0.86 (0.60)	<0.001
**Perceived Barrier (α = 0.79)**					
I don’t trust COVID-19 vaccine because it was developed in a short period of time	0.33 (0.47)	0.78 (0.42)	0.15 (0.36)	0.69 (0.46)	<0.001
I think COVID-19 vaccine would change your DNA	0.68 (0.66)	1.16 (0.64)	0.45 (0.59)	0.91 (0.67)	<0.001
I am concerned about side effects or risks of the vaccine	1.11 (0.84)	1.78 (0.50)	0.74 (0.78)	1.70 (0.57)	<0.001
I think the side effects of the COVID-19 vaccine are very serious/could lead to death	0.72 (0.70)	1.45 (0.61)	0.48 (0.59)	1.24 (0.70)	<0.001
The COVID-19 vaccine will not succeed because the virus keeps changing	0.90 (0.72)	1.49 (0.64)	0.60 (0.64)	1.22 (0.68)	<0.001
**Cues to Action**					
I would be more comfortable getting the vaccine if I saw neighbors, community leaders, religious leaders, doctors, celebrities, politicians receive the vaccine	1.77 (0.55)	0.90 (0.80)	1.82 (0.50)	1.00 (0.80)	0.0293
**Social Norms**					
Most people I know are going to receive the vaccine	1.16 (0.76)	0.54 (0.58)	1.24 (0.68)	0.70 (0.63)	0.006
**Knowledge (total score 0–8)**	5.37 (2.12)	4.47 (2.13)	7.22 (1.23)	6.23 (1.81)	<0.001

Items scored 0 = disagree, 1 = unsure, 2 = agree. *p* < 0.01. * Chi-square.

**Table 3:** Knowledge, attitudes, and beliefs regarding the COVID-19 vaccines in Survey 1 (pre-vaccine rollout) and Survey 2 (post-vaccine rollout).

Knowledge				
		Agree	Unsure	Disagree
I have enough information about the COVID-19 vaccine and how it works	Survey 1	23.3%	30.1%	46.6%
	Survey 2	65.0%	19.5%	15.6%
I know that there are different COVID-19 vaccines	Survey 1	55.3%	18.8%	25.9%
	Survey 2	85.1%	8.4%	6.5%
I know that the COVID-19 vaccine is available for all nationalities and free of charge	Survey 1	51.8%	29.7%	18.5%
	Survey 2	89.4%	7.5%	3.1%
I am aware of the categories who are eligible to take the vaccine first	Survey 1	46.5%	31.4%	22.1%
	Survey 2	64.5%	23.8%	11.7%
The vaccine can be given to pregnant women	Survey 1	-	-	-
	Survey 2	27.9%	29.0%	43.2%
**Attitudes and Beliefs**		**Agree**	**Unsure**	**Disagree**
It is possible that I would accept a certain vaccine and refuse another	Survey 1	21.4%	42.4%	36.2%
	Survey 2	47.6%	29.8%	22.6%
Given the variety of vaccines and their evolution I would prefer to wait until they are proven to be safe	Survey 1	68.8%	16.1%	15.1%
	Survey 2	53.9%	16.3%	29.9%
I think there is a better way than the vaccine to fight COVID-19	Survey 1	23.4%	33.6%	42.9%
	Survey 2	15.8%	33.8%	50.4%
If I receive the vaccine, I will not need to wear a mask anymore	Survey 1	17.5%	26.6%	55.9%
	Survey 2	18.1%	20.4%	61.6%
If a person has an allergy, they should not receive the vaccine	Survey 1	42.8%	43.3%	13.9%
	Survey 2	39.7%	28.8%	31.5%
I believe that the Lebanese health system is capable of ensuring safe administration of the COVID-19 vaccine	Survey 1	36.5%	-	63.5%
	Survey 2	50.2%	-	49.8%

**Table 4:** Preference regarding vaccination sites among Lebanese nationals and refugees.

	Survey 1			Survey 2		
	Overall	Lebanese	Refugees	Overall	Lebanese	Refugees
I prefer to receive the vaccine in Ministry of Public Health vaccination centers	37.3%	35.0%	39.4%	58.7%	67.6%	50.9%
I prefer to receive the vaccine in a private clinic/hospital even if it’s not free	19.0%	22.0%	16.3%	19.2%	23.7%	15.3%
I prefer to receive the vaccine in a primary health care center or dispensary near me	32.7%	29.4%	35.6%	40.3%	44.0%	37.1%
I prefer to receive the vaccine if provided at my residence (street or home or camp)	-	-	-	29.3%	34.6%	24.7%

Percentages do not add to 100% as participants could select more than one option.

**Table 5:** Intention to receive vaccine by HBM domains, social norms, and vaccine knowledge in multivariable analysis.

	Survey 1		Survey 2	
	aOR (95% CI)	*p*	aOR (95% CI)	*p*
**Gender**		0.897		0.531
Female	Ref		Ref	
Male	0.99 (0.79–1.22)		1.06 (0.88–1.29)	
**Age Category**		0.563		0.049
18–30 years	Ref		Ref	
31–50 years	1.13 (0.89–1.45)		1.13 (0.90–1.43)	
≥51 years	1.13 (0.84–1.52)		1.37 (1.06–1.78)	
**Nationality**		0.427		<0.001
Lebanese	Ref		Ref	
Refugees	0.91 (0.73–1.14)		0.50 (0.41–0.60)	
**Perceived Susceptibility**	1.24 (1.13–1.36)	<0.001	1.39 (1.28–1.51)	<0.001
**Perceived Severity**	0.93 (0.73–1.19)	0.582	1.27 (1.00–1.61)	0.052
**Perceived Benefits**	3.30 (2.89–3.81)	<0.001	2.26 (2.02–2.53)	<0.001
**Perceived Barriers**	0.69 (0.65–0.73)	<0.001	0.70 (0.66–0.74)	<0.001
**Cues to Action**	2.67 (2.26–3.16)	<0.001	2.55 (2.20–2.95)	<0.001
**Social Norms**	1.64 (1.38–1.95)	<0.001	1.31 (1.13–1.53)	<0.001
**Knowledge**	1.08 (1.03–1.15)	0.004	1.05 (0.98–1.12)	0.168
	Pseudo R^2^	0.642		0.684
	AUC 0.929	VIF 3.04	AUC 0.933	VIF 4.92

## Data Availability

The de-identified dataset is available upon reasonable request to the corresponding author.

## Supplementary Materials

The following supporting information can be downloaded at: https://www.mdpi.com/article/10.3390/vaccines10091533/s1, Table S1: Intention to receive vaccine by HBM domains, social norms and vaccine knowledge in univariate analysis; Table S2: Participant responses to HBM domain questions, stratified by na-tionality (Lebanese vs. refugees) in survey 1 (February 2021); Table S3: Participant responses to HBM domain questions, stratified by nationality (Lebanese vs. refugees) in survey 2 (June 2021).
