# Supplementary material for: Variations in COVID-19 Vaccine Attitudes and Acceptance among Refugees and Lebanese Nationals Pre- and Post-Vaccine Rollout in Lebanon
Source: Vaccines (Basel). 2022 Sep 15;10(9):1533. doi: 10.3390/vaccines10091533 (PMC9501461; doi:10.3390/vaccines10091533)
Supplement: Supplementary file 1 [file vaccines-10-01533-s001.zip › vaccines-1904858-supplementary.pdf]

Supplementary Table S1. Intention to receive vaccine by HBM domains, social norms and vaccine knowledge in univariate analysis.

	Survey 1		Survey 2	
	**OR (95% CI)**	**p**	**OR (95% CI)**	**p**
**Gender**		0.189		0.032
Female	Ref		Ref	
Male	1.10 (0.95-1.28)		1.14 (1.0-1.29)	
**Age Category**		0.175		0.004
18-30 years	Ref		Ref	
31-50 years	1.07 (0.91-1.27)		1.25 (1.07-1.45)	
≥51 years	1.20 (0.99-1.45)		1.97 (1.68-2.32)	
**Nationality**		0.043		<0.001
Lebanese	Ref		Ref	
Refugees	0.86 (0.74-1.00)		0.36 (0.32-0.41)	
**Perceived Susceptibility**	1.80 (1.70-1.91)	<0.001	2.25 (2.13-2.37)	<0.001
**Perceived Severity**	2.66 (2.27-3.10)	<0.001	5.34 (4.60-6.21)	<0.001
**Perceived Benefits**	5.85 (5.17-6.63)	<0.001	4.47 (4.10-4.87)	<0.001
**Perceived Barriers**	0.51 (0.49-0.54)	<0.001	0.47 (0.45-0.49)	<0.001
**Cues to Action**	5.77 (5.00-6.65)	<0.001	5.96 (5.27-6.74)	<0.001
**Social Norms**	4.06 (3.60-4.59)	<0.001	3.28 (2.96-3.63)	<0.001
**Knowledge**	1.23 (1.18-1.27)	<0.001	1.54 (1.47-1.62)	<0.001

Supplementary Table S2. Participant responses to HBM domain questions, stratified by nationality (Lebanese vs. refugees) in survey 1 (February 2021).

	**Lebanese n(%) N=1,850**			**Refugees n(%) N=2,077**			**p**
**Domain**	**Yes**	**Unsure**	**No**	**Yes**	**Unsure**	**No**	
**Perceived Susceptibility**							
I do not need to receive the vaccine because I have good health	713 (38.5)	467 (25.2)	670 (36.2)	1068 (51.4)	467 (22.5)	542 (26.1)	<0.001
I do not need to receive the vaccine if had been infected with COVID-19 and recovered	521 (28.2)	595 (32.2)	734 (39.7)	729 (35.1)	677 (32.6)	671 (32.3)	<0.001
**Perceived Severity**							
I am not at risk of severe complications of COVID-19 so I will not take the vaccine	783 (42.3)	-	1067 (57.7)	1114 (53.6)	-	963 (46.4)	<0.001
**Perceived Benefit**							
I think the COVID-19 vaccine is safe	291 (15.7)	924 (50.0)	635 (34.3)	317 (15.3)	994 (47.9)	766 (36.9)	0.246
I think the COVID-19 vaccine is effective	311 (16.8)	1005 (54.3)	534 (28.9)	307 (14.8)	1130 (54.4)	640 (30.8)	0.149
**Perceived Barrier**							
I don't trust COVID-19 vaccine because it was developed in a short period of time	1206 (65.2)	-	644 (34.8)	1418 (68.3)	-	659 (31.7)	0.041
I think COVID-19 vaccine would change your DNA	522 (28.2)	894 (48.2)	434 (23.5)	465 (22.4)	1227 (59.1)	385 (18.5)	<0.001
I am concerned about side effects or risks of the vaccine	1307 (70.7)	351 (19.0)	192 (10.4)	1497 (72.1)	376 (18.1)	204 (9.8)	0.613
I think the side effects of the COVID-19 vaccine are very serious/could lead to death	758 (41.0)	792 (42.8)	300 (16.2)	900 (43.3)	873 (42.0)	304 (14.6)	0.221
The COVID-19 vaccine will not succeed because the virus keeps changing	891 (48.2)	691 (37.4)	268 (14.5)	996 (48.0)	814 (39.2)	267 (12.9)	0.249
**Cues to Action**							
I would be more comfortable getting the vaccine if I saw neighbors, community leaders, religious leaders, doctors, celebrities, politicians receive the vaccine	727 (39.3)	552 (29.8)	571 (30.9)	896 (43.1)	563 (27.1)	618 (29.8)	0.039
**Social Norms**							
Most people I know are going to receive the vaccine	230 (12.4)	766 (41.4)	854 (46.2)	263 (12.7)	951 (45.8)	863 (41.6)	0.011

Supplementary Table S3. Participant responses to HBM domain questions, stratified by nationality (Lebanese vs. refugees) in survey 2 (June 2021).

	Lebanese n(%) N=1,950			Refugees n(%) N=2,224			
**Domain**	**Yes**	**Unsure**	**No**	**Yes**	**Unsure**	**No**	
**Perceived Susceptibility**							
I do not need to receive the vaccine because I have good health	466 (23.9)	285 (14.6)	1199 (61.5)	938 (42.2)	357 (16.1)	929 (41.8)	<0.001
I do not need to receive the vaccine if had been infected with COVID-19 and recovered	356 (18.3)	394 (20.2)	1200 (61.5)	601 (27.0)	572 (25.7)	1051 (47.3)	<0.001
**Perceived Severity**							
I am not at risk of severe complications of COVID-19 so I will not take the vaccine	494 (25.3)	-	1456 (74.7)	920 (41.4)	-	1304 (58.6)	<0.001
**Perceived Benefit**							
I think the COVID-19 vaccine is safe	907 (46.5)	802 (41.1)	241 (12.4)	691 (31.1)	1113 (50.0)	420 (18.9)	<0.001
I think the COVID-19 vaccine is effective	837 (42.9)	884 (45.3)	229 (11.7)	652 (29.3)	1181 (53.1)	391 (17.6)	<0.001
**Perceived Barrier**							
I don't trust COVID-19 vaccine because it was developed in a short period of time	718 (36.8)	-	1232 (63.2)	1177 (52.9)	-	1047 (47.1)	<0.001
I think COVID-19 vaccine would change your DNA	199 (10.2)	940 (43.1)	911 (46.7)	323 (14.5)	1059 (47.6)	842 (37.9)	<0.001
I am concerned about side effects or risks of the vaccine	768 (39.4)	565 (29.0)	617 (31.6)	1366 (61.4)	482 (21.7)	376 (16.9)	<0.001
I think the side effects of the COVID-19 vaccine are very serious/could lead to death	342 (17.5)	835 (42.8)	773 (39.6)	656 (29.5)	931 (41.9)	637 (28.6)	<0.001
The COVID-19 vaccine will not succeed because the virus keeps changing	401 (20.6)	875 (44.9)	674 (34.6)	605 (27.2)	1055 (47.4)	564 (25.4)	<0.001
**Cues to Action**							
I would be more comfortable getting the vaccine if I saw neighbors, community leaders, religious leaders, doctors, celebrities, politicians receive the vaccine	1205 (61.8)	414 (21.2)	331 (17.0)	1148 (51.6)	581 (26.1)	495 (22.3)	<0.001
**Social Norms**							
Most people I know are going to receive the vaccine	595 (30.5)	921 (47.2)	434 (22.3)	325 (14.6)	1162 (52.3)	737 (33.1)	<0.001
